# The Characteristics and Mortality of Osteoporosis, Osteomyelitis, or Rheumatoid Arthritis in the Diabetes Population: A Retrospective Study

**DOI:** 10.1155/2020/8821978

**Published:** 2020-11-07

**Authors:** Jin-Feng Huang, Qi-Nan Wu, Xuan-Qi Zheng, Xiao-Lei Sun, Chen-Yu Wu, Xiao-Bing Wang, Chen-Wei Wu, Bin Wang, Xiang-Yang Wang, Michael Bergman, Ai-Min Wu

**Affiliations:** ^1^Department of Orthopaedics, The Second Affiliated Hospital and Yuying Children's Hospital of Wenzhou Medical University, Wenzhou, Zhejiang 325027, China; ^2^The Second School of Medicine, Wenzhou Medical University, Wenzhou, Zhejiang 325027, China; ^3^Endocrinology and Nephrology Department, Chongqing University Cancer Hospital and Chongqing Cancer Institute and Chongqing Cancer Hospital, Chongqing, China; ^4^Department of Orthopaedics, Tianjin Hospital, Tianjin 300210, China; ^5^Department of Rheumatology, The First Affiliated Hospital of Wenzhou Medical University, Wenzhou, China; ^6^Diabetes Center and Department of Endocrinology, The Second Affiliated Hospital and Yuying Children's Hospital of Wenzhou Medical University, Wenzhou, China; ^7^Department of Sports Medicine and Adult Reconstruction Surgery, Nanjing Drum Tower Hospital, The Affiliated Hospital of Nanjing University Medical School, Nanjing 210009, China; ^8^NYU Grossman School of Medicine, NYU Langone Diabetes Prevention Program, New York, NY 10016, USA

## Abstract

**Background:**

Patients with diabetes mellitus are prone to develop osteoporosis, osteomyelitis, or rheumatoid arthritis (RA). Furthermore, the presence of these complications in those with diabetes may lead to higher mortality. The aim of our study was to assess characteristics and mortality of osteoporosis, osteomyelitis, or rheumatoid arthritis in individuals with diabetes.

**Methods:**

We analyzed osteoporosis, osteomyelitis, and RA deaths associated with diabetes from 1999–2017 using the CDC WONDER system (CDC WONDER; https://wonder.cdc.gov). We used *ICD-10* codes to categorize the underlying and contributing causes of death. Crude mortality rates (CMR) and age-adjusted mortality rates (AAMR) per 1,000,000 person-years were calculated.

**Results:**

The AAMR for osteoporosis in the population with diabetes was significantly higher in females (AAMR: 4.17, 95% CI: 4.10–4.24) than in males (AAMR: 1.12, 95% CI: 1.07–1.16). Deaths due to osteoporosis increased gradually from 1999, peaked in 2003 (AAMR: 3.78, 95% CI: 3.55–4.00), and reached a nadir in 2016 (AAMR: 2.32, 95% CI: 2.15–2.48). The AAMR for RA associated with diabetes was slightly higher in females (AAMR: 4.04, 95% CI: 3.98–4.11) than in males (AAMR: 2.45, 95% CI: 2.39–2.51). The mortality rate due to RA increased slightly from 1999 (AAMR: 3.18, 95% CI: 2.97–3.39) to 2017 (AAMR: 3.20, 95% CI: 3.02–3.38). The AAMR for osteomyelitis associated with diabetes was higher in males (AAMR: 4.36, 95% CI: 4.28–4.44) than in females (AAMR: 2.31, 95% CI: 2.26–2.36). From 1999 to 2017, the AAMR from osteomyelitis in this population was 2.63 (95% CI: 2.44–2.82) per 1,000,000 person-years in 1999 and 4.25 (95% CI: 4.05–4.46) per 1,000,000 person-years in 2017.

**Conclusions:**

We found an increase in the age-adjusted mortality rates of RA and osteomyelitis and a decrease of osteoporosis associated with diabetes from 1999 to 2017. We suggest that increased attention should therefore be given to these diseases in the population with diabetes, especially in efforts to develop preventative and treatment strategies.

## 1. Introduction

About 451 million people worldwide are affected by diabetes representing a global prevalence of 8.8% [1] which has increased in the past 50 years [2]. People with diabetes have a greater risk of life-threatening health problems which can result in higher medical costs, reduced quality of life, and increased mortality [3]. Diabetes can increase the risks of cardiovascular diseases, infection, cancer [4–8], and the development of musculoskeletal conditions such as osteoporosis, osteomyelitis, and rheumatoid arthritis (RA) [9–12] which may be closely associated with and have a higher prevalence and mortality in diabetes. However, the specific burden of mortality from these three diseases associated with diabetes mellitus is unknown.

As multimorbidities may increase the burden in a given individual [13], there has been increased research and clinical interests regarding comorbidities in the last decades [14–16]. Diabetes is a systemic disease commonly coexisting with other entities [17]. Furthermore, comorbidities may reduce physical function, decrease quality of life, and increase mortality [18]. Therefore, understanding the characteristics and specific mortality rates of diabetes associated with osteoporosis, osteomyelitis, or RA is important for prevention and treatment. To the best of our knowledge, this topic, focused on the mortality of diabetes mellitus in association with comorbid musculoskeletal diseases, has not been previously studied. The primary purpose of this study, therefore, was to assess the characteristics, trends, and mortality of osteoporosis, osteomyelitis, or RA in the diabetes population from 1999 to 2017.

## 2. Materials and Methods

Mortality data of osteoporosis, osteomyelitis, and RA associated with or without diabetes were obtained from the National Center for Health Statistics multiple cause of death for 1999–2017 from the U.S. CDC WONDER system (CDC WONDER; https://wonder.cdc.gov) [19]. The National Vital Statistics System (NVSS) provided mortality data from death certificates filed in the 50 states and the District of Columbia, in CDC WONDER [20]. The study period analyzed in this project represented all years of mortality data available at the time of analysis using the International Classification of Disease, Tenth Revision (*ICD-10*) code set.

The World Health Organization (WHO) has defined the underlying cause of death as the disease or injury that initiated the series of events leading directly to death and a contributing cause of death as a disease or injury that can be considered a contributing factor leading to death [21]. We used *ICD-10* codes to categorize the underlying and contributing causes of death as has been done previously [22]. We defined diabetes mellitus as *ICD-10* codes E10-E14, [23] osteoporosis as *ICD-10* codes M80-M82, osteomyelitis as *ICD-10* code M86, and RA as *ICD-10* codes M05-M06.9 and M08.0-M08.89 [24].

### 2.1. Statistical Analyses

Crude mortality rates (CMR) were calculated as deaths per 1,000,000 person-years. Age-adjusted mortality rates (AAMR) per 1,000,000 person-years were calculated for a 2000 US Standard population as designated by CDC WONDER [25, 26] (the specific calculation method is shown in Supplementary Materials).

Further analysis of unique individuals was conducted by sorting deaths by factors such as age groups (<55 years, 55–64 years, 65–74 years, 75–84 years, and ≥85 years), sex, race, region, and year of death. For individuals >65 years old, we further studied the trend of AAMR sorted by regions, race and sex, and states from 1999 to 2017. The percent of change in CMR or AAMR from 1999 to 2017 was calculated to show the increase or decrease in mortality. We also reported 95% confidence intervals (CIs) and standard errors (SEs) for CMR and AAMR. All statistical analyses were conducted with SPSS software (version 18, IBM Corp., USA).

## 3. Results

### 3.1. Mortality of Osteoporosis Associated with or without Diabetes

From 1999–2017, osteoporosis associated with diabetes led to 18,428 deaths, while diabetes leading 1,399,943 deaths and osteoporosis resulted in 25,209 deaths. The AAMR of osteoporosis associated diabetes was 3.01 per 1,000,000 person-years (95% CI: 2.96–3.05).

The AAMR for osteoporosis associated diabetes was significantly higher in females (AAMR: 4.17, 95% CI: 4.10–4.24) than in males (AAMR: 1.12, 95% CI: 1.07–1.16) (Table 1). The mortality rate for osteoporosis associated with diabetes increased with age. AAMR was lowest in the black or African (AAMR: 1.60, 95% CI: 1.48–1.71) populations and the Northeast region (AAMR: 2.15, 95% CI: 2.07–2.23). The AAMR of osteoporosis without diabetes was much higher in females (AAMR: 66.19, 95% CI: 65.89–66.48) and populations older than 85 years (1,374.22, 95% CI: 1,366.94–1,381.50).

**Figure 1:**
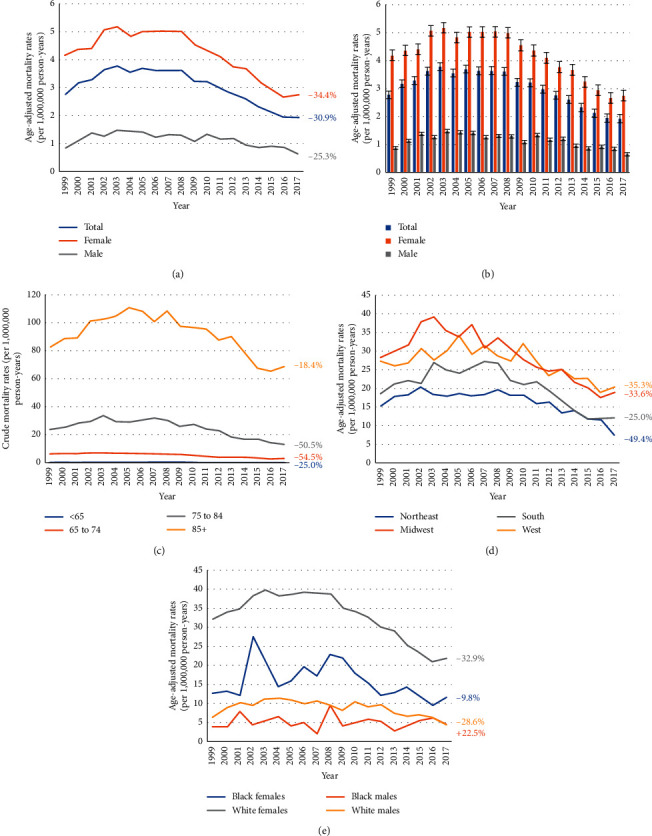
1999–2017 US age-adjusted mortality rates due to osteoporosis and diabetes for gender groups (a, b) and age groups (c). Age-adjusted mortality rates due to osteoporosis and diabetes among patients 65 years or older for census region groups (d) and both race and sex groups (e).

Deaths due to osteoporosis associated with diabetes increased gradually from 1999, peaked in 2003 (AAMR: 3.78, 95% CI: 3.55–4.00), and reached a nadir in 2016 (AAMR: 2.32, 95% CI: 2.15–2.48) (Figures 1(a) and 1(b) and Table S1). The AAMR of osteoporosis without diabetes decreased 59.50% from 1999 to 2017 (Table S1). Mortality decreased 25.0%, 54.5%, 50.5%, and 18.4% in the <65, 65 to 74, 75 to 84, and 85+ age groups, respectively (Figure 1(c)). In patients older than 65 years, the percent of deaths due to osteoporosis associated with diabetes decreased 35.3%, 33.6%, 25.0%, and 49.4% in the West, Midwest, South, and Northeast regions, respectively (Figure 1(d)). The percent of deaths decreased 32.9%, 9.8%, and 28.6% in white females, black females, and white males, respectively, while increasing 22.5% in black males (Figure 1(e)).

### 3.2. Mortality of RA Associated with Diabetes

From 1999–2017 years, RA associated with diabetes was reported as a contributing cause of death in 20,584 individuals nationwide. The AAMR from RA associated with diabetes was 3.35 per 1,000,000 person-years (95% CI: 3.31–3.40), compared to 7.67 (95% CI: 7.60–7.74) for osteomyelitis as a leading cause of death. As shown in Table 2, the AAMR for RA associated with diabetes was slightly higher in females (AAMR: 4.04, 95% CI: 3.98–4.11) than in males (AAMR: 2.45, 95% CI: 2.39–2.51). The mortality rate for RA associated with diabetes was 13.53 (95% CI: 13.18–13.88), 29.37 (95% CI: 28.70–30.04), and 39.49 (95% CI: 38.25–40.72) in the 65–74, 75–84, and 85+ age groups, respectively. Moreover, AAMR for RA associated with diabetes was lowest in Asians or in the Pacific Islander (AAMR: 1.88, 95% CI: 1.69–2.07) and the Northeast region (AAMR: 2.48, 95% CI: 2.39–2.57).

**Figure 2:**
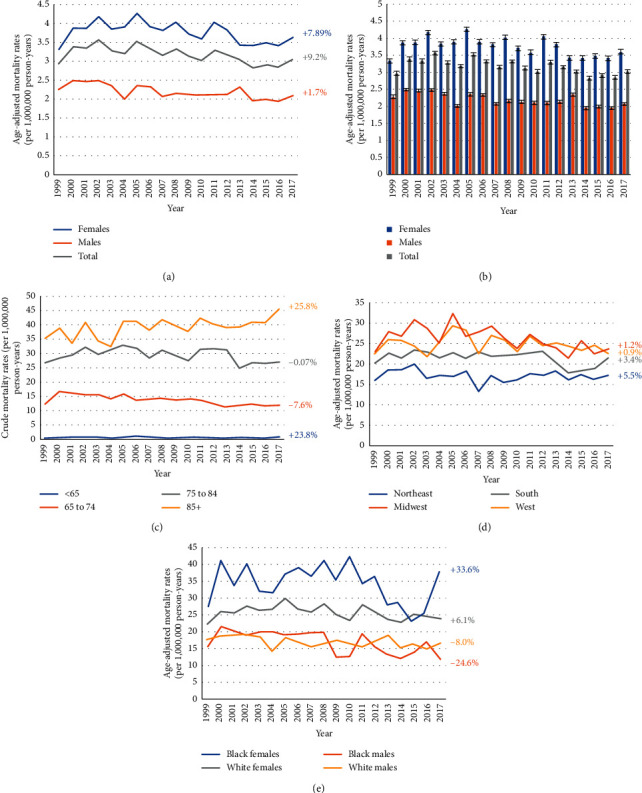
1999–2017 US age-adjusted mortality rates due to rheumatoid arthritis and diabetes for gender groups (a, b) and age groups (c). Age-adjusted mortality rates due to osteoporosis and diabetes among patients 65 years or older for census region groups (d) and both race and sex groups (e).

In general, death rates due to RA associated with diabetes slightly increased from 1999 (AAMR: 3.18, 95% CI: 2.97–3.39) to 2017 (AAMR: 3.20, 95% CI: 3.02–3.38) (Figures 2(a) and 2(b) and Table S2). The AAMR of RA without diabetes decreased 36.55% from 1999 to 2017 (Table S2), The percent mortality due to RA associated with diabetes increased in those <65 and 85+ years (23.8% and 25.8%, respectively) (Figure 2(c)). The change in mortality rate was relatively stable for the different census regions in patients older than 65 years (Figure 2(d)). However, in those older than 65 years, the percent of AAMR increased 33.6% and 6.1% in black females and white females, respectively, and decreased 24.6% and 8.0%, respectively, in black males and while males (Figure 2(e)).

### 3.3. Mortality of Osteomyelitis Associated with Diabetes

**Figure 3:**
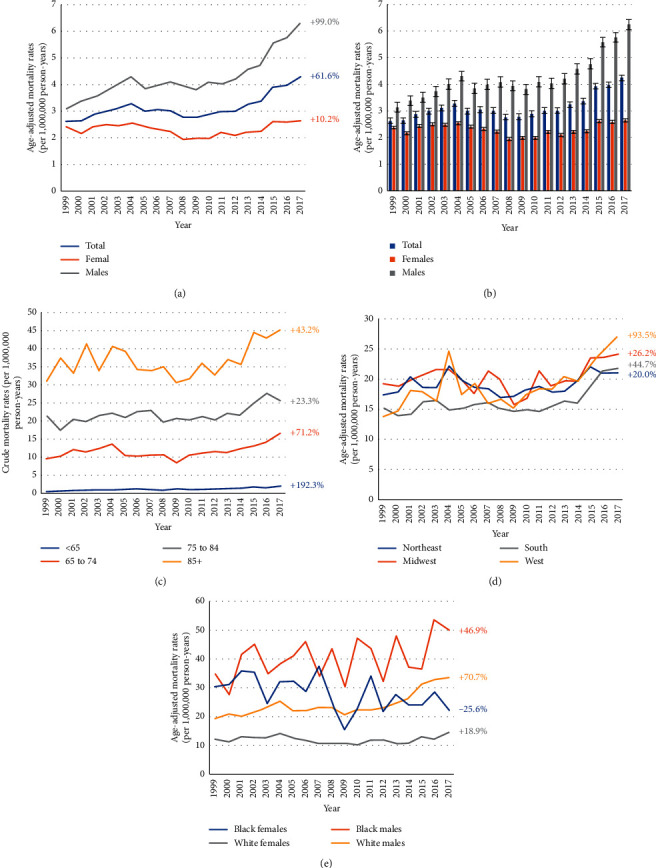
1999–2017 US age-adjusted mortality rates due to osteomyelitis and diabetes for the gender groups (a, b) and age groups (c). Age-adjusted mortality rates due to osteoporosis and diabetes among patients 65 years or older for census region groups (d) and both race and sex groups (e).

Osteomyelitis associated with diabetes was reported as a contributing cause of death in 19,726 individuals. The AAMR from osteomyelitis associated with diabetes was 2.63 per 1,000,000 person-years in 1999 (95% CI: 2.44–2.82) and 4.25 per 1,000,000 person-years in 2017 (95% CI: 4.05–4.46) (Table S3). While the AAMR of osteomyelitis without diabetes increased 53.04% from 1999 to 2017 (Table S3), the AAMR for osteomyelitis associated with diabetes was clearly higher in males (AAMR: 4.36, 95% CI: 4.28–4.44) than in females (AAMR: 2.31, 95% CI: 2.26–2.36) (Table 3). The mortality rate was 6.29 (95% CI: 5.50–7.09), 5.68 (95% CI: 5.48–5.88), 2.98 (95% CI: 2.93–3.02), and 1.41 (95% CI: 1.26–1.57) in American Indian, black or African American, white and Asian, or Pacific Islander, respectively. The crude mortality rate of osteomyelitis associated with diabetes increased with age (<55 years: 0.50, 95% CI: 0.48–0.52; 55 to 64 years: 5.49, 95% CI: 5.30–5.67; 65 to 74 years: 11.77, 95% CI: 11.44–12.10; 75 to 84 years: 21.82, 95% CI: 21.25–22.40; and 85+ years: 36.86, 95% CI: 35.67–38.05). AAMR of osteomyelitis associated with diabetes was highest in American Indian (AAMR: 6.29, 95% CI: 5.50–7.09) and lowest in Asians or in the Pacific Islander (AAMR: 1.41, 95% CI: 1.26–1.57). AAMR of osteomyelitis associated with diabetes was relatively similar in the four census regions (Northeast: 3.09, 95% CI: 2.99–3.19; Midwest: 3.44, 95% CI: 3.34–3.54; South: 2.95, 95% CI: 2.88–3.02; West: 3.37, 95% CI: 3.27–3.47). After mortality data were stratified by age, race, gender, and years from 1999 to 2017, we found that AAMR among males increased 99.0%, which was much higher than in females (10.2%) (Figures 3(a) and 3(b)).

The AAMR due to osteomyelitis associated with diabetes increased 192.3%, 71.2%, 23.3%, and 43.2% in the <65, 65 to 74, 75 to 84, and 85+ age groups, respectively (Figure 3(c)). In patients older than 65 years, the percent of deaths due to osteomyelitis associated with diabetes largely increased in the West (93.5%) (Figure 3(d)). For patients older than 65 years, the percent of deaths increased 70.7%, 46.9%, and 18.9% in white males, black males, and white females, respectively. However, the percent of deaths decreased 25.6% in black females (Figure 3(e)).

### 3.4. Mortality of Osteoporosis, Osteomyelitis, and RA Associated with Diabetes by States

**Figure 4:**
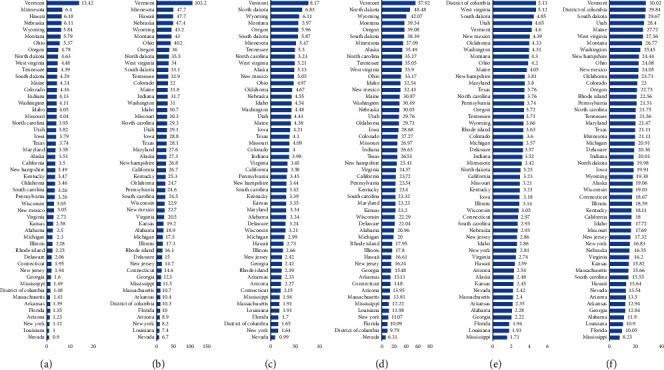
Age-adjusted mortality rates due to osteoporosis and diabetes among the general population (a) and 65 years or older population (b) for different states. Age-adjusted mortality rates due to rheumatoid arthritis and diabetes for the general population (c) and 65 years or older population (d) for different states. Age-adjusted mortality rates due to osteomyelitis and diabetes for the general population (e) and 65 years or older population (f) for different states.

Figure 4 shows different AAMR of osteoporosis, osteomyelitis, and RA associated with diabetes in different states, and the mortality differs considerably.

## 4. Discussion

In this study, 18,428 deaths attributable to osteoporosis associated with diabetes, 19,726 deaths attributable to osteomyelitis associated with diabetes, and 20,584 deaths attributable to RA associated with diabetes were reported in the United States between 1999 and 2017, each significantly affects the elderly (>65 years) population. Notably, the differences in reported mortality rates related to sex, age, race, and census regions may reflect different pathophysiological etiologies requiring further investigation.

### 4.1. Mortality of Osteoporosis Associated with Diabetes

Previous studies have shown that diabetes and osteoporosis are both chronic diseases which might lead to severe mortality [27, 28]. Overall, AAMR of osteoporosis associated with diabetes decreased from 2.91 in 1999 to 2.33 in 2017. A steady increase in the mortality rate was seen from 1999 to 2003, but in 2004, the rates declined throughout the remainder of the study period. The mortality rate was shown to decrease faster in older cohorts, especially women, similar to the present findings [29]. The mortality rate for those >65+ years demonstrated an inverted U-shape from 1999 to 2017 for the four census regions. It is important to note that while mortality decreased from 1999 to 2017 in white females, black females, and white males older than 65 years, the mortality rate increased in black males. Furthermore, the mortality of osteoporosis related with diabetes was highest in white females older than 65 years. Previous studies have shown that the mortality and incidence of osteoporosis largely occurred in women, especially in white women, [30–32] which is consistent with our data.

Fragility fractures were one of the most common reasons leading to excess death in both type 1 diabetes (T1D) and type 2 diabetes (T2D) [33, 34]. Sehgal et al. demonstrated that the incidence of hospitalizations from osteoporotic fractures declined in both females and males over the age of 50 years [35]. This may explain the rapid decline in the mortality rate after 2009. In recent decades, the main treatment of bone disorders associated with diabetes consisted of medications to control diabetes and vitamin D supplementation [36]. However, there is little evidence supporting treatment regimens for diabetes-associated bone disorders [36, 37]. Though the low-turnover state in diabetes might hamper the effect of antiresorptive drugs such as bisphosphonates, previous studies showed that diabetes does not seem diminish the efficacy of these agents on bone mineral density (BMD) and their potential to reduce fractures [38–41]. Additionally, previous studies have noted a decline in bisphosphonate prescriptions between 2007 and 2008 in the US and an increase in Internet searches and media reports about the safety of oral bisphosphonate prescriptions [42, 43]. Coincidently, the mortality rate peaked between 2007 and 2008 and has been dropping rapidly after 2009. Trends in medical care may potentially explain the change in mortality of osteoporosis associated with diabetes. However, there are little data to show the effectiveness of antiresorptive medications in diabetes associated with osteoporosis mainly relying on anecdotal experience and limited case reports [36, 37]. Therefore, more large population-based studies should be conducted to evaluate the effect of antiresorptive drugs for treating osteoporosis in diabetes, and specific guidelines need to be developed accordingly.

### 4.2. Mortality of RA Associated with Diabetes

Diabetes is an important risk factor for the higher mortality observed in patients with RA [44–46]. In general, AAMR of RA associated with diabetes did not change significantly from 1999 to 2017. However, the mortality of individuals over 85 years increased considerably while it decreased in the 65 to 74 and 75 to 84-year age groups. Bandyopadhyay et al. pointed out that the proportion of RA patients with comorbidities, especially cardiovascular disease, increased from 2005 to 2014 [47]. It is worthwhile noting that both RA and diabetes increase the risk of cardiovascular disease [48, 49]. These results may also explain the gradual increase in the AAMR in those over 85 years having a high incidence of cardiovascular disease. Therefore, management of comorbidities, especially cardiovascular disease, in patients with RA and diabetes is vital for reducing mortality of RA associated with diabetes [50].

Mortality was highest in black females with the largest rise noted from 2015 to 2017. The proinflammatory cytokine interleukin-6 IL-6-174 G/G genotype was found to be about 36.5 times more frequent in blacks compared to the white population [51]. The presence of a high-grade systemic inflammatory state may therefore explain the increased prevalence of cardiovascular disease in RA [52–55] and the higher mortality rate in black females. Additional studies should focus on race-specific therapeutic effects of IL-6 blockade such as tocilizumab.

### 4.3. Mortality of Osteomyelitis Associated with Diabetes

Patient with diabetes may have a 15–25% lifetime risk of developing a foot ulcer with 20% of infections progressing to osteomyelitis [56–59] which is associated with excess mortality [60, 61]. The present results showed the mortality from osteomyelitis associated with diabetes significantly increased from 1999 to 2017, especially in men. Kremers et al. pointed out that the incidence of diabetes associated with osteomyelitis increased in the US with a higher prevalence in males than in females [62]. A more recent study demonstrated that the incidence of osteomyelitis more than doubled between 2008 and 2017 [63]. These results may explain the increased mortality rate with time in the present study. In addition, Yoshimoto et al. indicated that the increase in the incidence of osteomyelitis was associated with aging [64], and results were also found in the present study. The AAMR increased significantly in different age groups.

Previous studies have shown that different clinical practice guidelines for diabetes-related osteomyelitis may result in conflicting recommendations, reducing the effectiveness of treatment [65, 66]. However, there is no generally agreed-upon treatment protocol for diabetes associated with osteomyelitis, making its management more difficult than in other diabetes-related conditions [67]. In last decades, the treatment of diabetes-related osteomyelitis consisted of antibiotics and resecting the necrotic and infected bone [68]. However, these methods have limitations which include a high risk of recurrent infections and ulceration, toxicity and adverse effects due to prolonged antibiotic administration, and development of bacterial resistance [68, 69]. These sequelae may lead to treatment failure and even death. Therefore, improving the treatment outcomes of diabetes associated with osteomyelitis requires the development of more effective clinical practice guidelines and treatment protocols [66].

We also found that the AAMR of osteomyelitis with diabetes increased more than osteomyelitis without diabetes. The decreased trend of AAMR in osteoporosis or RA among diabetes populations was significantly less than in nondiabetes populations from 1999 to 2017. These results showed that control of diabetes is vital in patients with many comorbidities.

## 5. Limitations

Our study has some limitations. First, as we used vital statistics data, there may have been potential deaths missed or incorrectly allocated. Second, since the diagnosis of osteoporosis may be more likely in older age groups, there may have been inherent coding biases. Third, as data could not be analyzed on the individual level, further analysis of specific factors which may associated with prognosis could not be performed.

## 6. Conclusions

The present study identified several important factors regarding age, gender, race, and census regions related to mortality of osteoporosis, osteomyelitis, and RA in the diabetes population. We found an increase in age-adjusted mortality rates of RA and osteomyelitis and a decrease in osteoporosis associated with diabetes from 1999 to 2017. Osteoporosis, osteomyelitis, or rheumatoid arthritis in the population with diabetes is therapeutically challenging and may increase the risk of death. Therefore, increased attention should be paid to these entities in diabetes, especially for undertaking preventative and treatment strategies.

## Figures and Tables

**Table 1:** Demographics of individuals with mortality from osteoporosis with or without diabetes.

	Both diabetes, osteoporosis, *N* (%)	Crude rate per 1,000,000	Age-adjusted rate per 1,000,000	Osteoporosis without diabetes, *N* (%)	Crude rate per 1,000,000	Age-adjusted rate per 1,000,000	Standard US population in 2000
Total	18,428 (100%)	3.20 (3.15–3.24)	3.01 (2.96–3.05)	219,744 (100%)	38.14 (37.98–38.30)	35.56 (35.41–35.71)	5,761,465,567

Sex							
Female	15,757 (85.51%)	5.38 (5.30–5.46)	4.17 (4.10–4.24)	193,877 (88.23%)	66.19 (65.89–66.48)	49.56 (49.33–49.78)	2,929,154,929
Male	2,671 (14.49%)	0.94 (0.91–0.98)	1.12 (1.07–1.16)	25,867 (11.77%)	9.13 (9.02–9.24)	11.34 (11.20–11.47)	2,832,310,638

Race							
American Indian	103 (0.56%)	1.39 (1.12–1.66)	3.08 (2.46–3.71)	614 (0.28%)	8.30 (7.65–8.96)	20.07 (18.43–21.70)	73,938,616
Asian or Pacific Islander	602 (3.27%)	1.97 (1.81–2.12)	3.20 (2.94–3.46)	4,016 (1.83%)	13.12 (12.71–13.53)	21.75 (21.07–22.42)	306,084,526
Black or African American	794 (4.31%)	1.02 (0.95–1.09)	1.60 (1.48–1.71)	4,584 (2.09%)	5.88 (5.71–6.05)	9.33 (9.06–9.60)	778,991,453
White	16,929 (91.87%)	3.68 (3.62–3.73)	3.14 (3.10–3.19)	210,530 (95.81%)	45.74 (45.55–45.94)	38.44 (38.27–38.60)	4,602,450,972

Age groups							
<55 years	289 (1.57%)	0.07 (0.06–0.07)	—	1,648 (0.75%)	0.38 (0.36–0.40)	—	4,355,837,726
55–64 years	693 (3.76%)	1.08 (1.00–1.16)	—	4,892 (2.23%)	7.65 (7.44–7.87)	—	639,299,997
65–74 years	2,229 (12.10%)	5.36 (5.14–5.58)	—	16,675 (7.59%)	40.09 (39.48–40.70)	—	415,933,194
75–84 years	6,179 (33.53%)	24.65 (24.03–25.26)	—	59,511 (27.08%)	237.39 (235.48–239.30)	—	250,688,640
85+ years	9,041 (49.06%)	90.68 (88.81–92.55)	—	137,018 (62.35%)	1,374.22 (1,366.94–1,381.50)	—	99,706,010

Census region							
Northeast	2,725 (14.79%)	2.61 (2.51–2.71)	2.15 (2.07–2.23)	34,608 (15.75%)	33.12 (32.77–33.46)	26.73(26.45–27.02)	1,045,051,171
Midwest	5,430 (29.47%)	4.31 (4.19–4.42)	3.80 (3.70–3.91)	65,278 (29.71%)	51.76 (51.36–52.16)	44.91(44.56–45.25)	1,261,166,722
South	5,793 (31.44%)	2.73 (2.66–2.80)	2.68 (2.61–2.75)	66,024 (30.05%)	31.13 (30.89–31.37)	30.73(30.50–30.97)	2,120,820,931
West	4,480 (24.31%)	3.36 (3.26–3.46)	3.52 (3.42–3.62)	53,834 (24.50%)	40.34 (40.00–40.68)	41.94(41.59–42.30)	1,334,426,743

**Table 2:** Demographics of individuals with mortality from rheumatoid arthritis with or without diabetes.

	Both diabetes and rheumatoid arthritis, *N* (%)	Crude rate per 1,000,000	Age-adjusted rate per 1,000,000	Rheumatoid arthritis without diabetes, *N* (%)	Crude rate per 1,000,000	Age-adjusted rate per 1,000,000	Standard US population in 2000
Total	20,584 (100%)	3.57 (3.52–3.62)	3.35 (3.31–3.40)	170,291 (100%)	29.56 (29.42–29.70)	27.92 (27.78–28.05)	5,761,465,567

Sex							
Female	14,245 (69.20%)	4.86 (4.78–4.94)	4.04 (3.98–4.11)	125,203 (73.52%)	42.74 (42.51–42.98)	34.96 (34.76–35.15)	2,929,154,929
Male	6,339 (30.80%)	2.24 (2.18–2.29)	2.45 (2.39–2.51)	45,088 (26.48%)	15.92 (15.77–16.07)	17.80 (17.64–17.97)	2,832,310,638

Race							
American Indian	324 (1.57%)	4.38 (3.90–4.86)	7.68 (6.79–8.57)	1,677 (0.98%)	22.68 (21.60–23.77)	41.51 (39.39–43.62)	73,938,616
Asian or Pacific Islander	400 (1.94%)	1.31 (1.18–1.43)	1.88 (1.69–2.07)	2,686 (1.58%)	8.78 (8.44–9.11)	12.95 (12.45–13.45)	306,084,526
Black or African American	2,253 (10.95%)	2.89 (2.77–3.01)	4.10 (3.93–4.28)	11,963 (7.03%)	15.36 (15.08–15.63)	22.07 (21.67–22.47)	778,991,453
White	17,607 (85.54%)	3.83 (3.77–3.88)	3.31 (3.26–3.36)	153,965 (90.41%)	33.45 (33.29–33.62)	28.96 (28.82–29.11)	4,602,450,972

Age groups							
<55 years	939 (4.56%)	0.22 (0.20–0.23)	—	7,244 (4.26%)	1.66 (1.62–1.70)	—	4,355,837,726
55–64 years	2,718 (13.20%)	4.25 (4.09–4.41)	—	16,674 (9.79%)	2.61 (2.57–2.65)	—	639,299,997
65–74 years	5,627 (27.34%)	13.53 (13.18–13.88)	—	37,834 (22.22%)	9.10 (9.00–9.19)	—	415,933,194
75–84 years	7,363 (35.77%)	29.37 (28.70–30.04)	—	63,557 (37.32%)	25.35 (25.16–25.55)	—	250,688,640
85+ years	3,937 (19.13%)	39.49 (38.25–40.72)	—	44,981 (26.41%)	45.11 (44.70–45.53)	—	99,706,010

Census region							
Northeast	3,002 (14.58%)	2.87 (2.77–2.98)	2.48 (2.39–2.57)	26,707 (15.68%)	25.56 (25.25–25.86)	21.74 (21.48–22.01)	1,045,051,171
Midwest	5,400 (26.23%)	4.28 (4.17–4.40)	3.86 (3.76–3.97)	45,460 (26.70%)	36.05 (35.71–36.38)	32.57 (32.27–32.88)	1,261,166,722
South	7,316 (35.54%)	3.45 (3.37–3.53)	3.26 (3.19–3.34)	57,349 (33.68%)	27.04 (26.82–27.26)	26.02 (25.81–26.24)	2,120,820,931
West	4,866 (23.64%)	3.65 (3.54–3.75)	3.74 (3.63–3.84)	40,775 (23.94%)	30.56 (30.26–30.85)	31.72 (31.41–32.03)	1,334,426,743

**Table 3:** Demographics of individuals with mortality from osteomyelitis with or without diabetes.

	Both diabetes and osteomyelitis, *N* (%)	Crude rate per 1,000,000	Age-adjusted rate per 1,000,000	Osteomyelitis without diabetes, *N* (%)	Crude rate per 1,000,000	Age-adjusted rate per 1,000,000	Standard US population in 2000
Total	19,726 (100%)	3.42 (3.38–3.47)	3.17 (3.13–3.22)	44,170 (100%)	7.67 (7.58–7.76)	7.17 (7.09–7.25)	5,761,465,567

Sex							
Female	8,192 (41.53%)	2.80 (2.74–2.86)	2.31 (2.26–2.36)	21,422 (48.50%)	7.31 (7.19–7.43)	5.82 (5.73–5.92)	2,929,154,929
Male	11,534 (58.47%)	4.07 (4.00–4.15)	4.36 (4.28–4.44)	22,748 (51.50%)	8.03 (7.91–8.16)	9.01 (8.86–9.15)	2,832,310,638

Race							
American Indian	274 (1.39%)	3.71 (3.27–4.14)	6.29 (5.50–7.09)	320 (0.72%)	4.32 (3.68–4.97)	8.01 (6.77–9.24)	73,938,616
Asian or Pacific Islander	324 (1.64%)	1.06 (0.94–1.17)	1.41 (1.26–1.57)	599 (1.36%)	1.96 (1.76–2.15)	2.84 (2.56–3.12)	306,084,526
Black or African American	3,290 (16.68%)	4.22 (4.08–4.37)	5.68 (5.48–5.88)	7,288 (16.50%)	9.36 (9.10–9.62)	13.38 (13.01–13.76)	778,991,453
White	15,838 (80.29%)	3.44 (3.39–3.49)	2.98 (2.93–3.02)	35,963 (81.42%)	7.82 (7.72–7.91)	6.70 (6.62–6.79)	4,602,450,972

Age groups							
<55 years	2,176 (11.03%)	0.50 (0.48–0.52)	—	3,732 (8.45%)	0.86 (0.82–0.89)	—	4,355,837,726
55–64 years	3,507 (17.78%)	5.49 (5.30–5.67)	—	5,093 (11.53%)	7.96 (7.68–8.25)	—	639,299,997
65–74 years	4,897 (24.83%)	11.77 (11.44–12.10)	—	7,884 (17.85%)	18.96 (18.43–19.49)	—	415,933,194
75–84 years	5,471 (27.73%)	21.82 (21.25–22.40)	—	12,648 (28.63%)	50.46 (49.40–51.51)	—	250,688,640
85+ years	3,675 (18.63%)	36.86 (35.67–38.05)	—	14,813 (33.54%)	148.57 (145.89–151.24)	—	99,706,010

Census region							
Northeast	3,789 (19.21%)	3.63 (3.51–3.74)	3.09 (2.99–3.19)	9,406 (21.29%)	9.0 (8.78–9.21)	7.60 (7.41–7.78)	1,045,051,171
Midwest	5,400 (27.38%)	3.82 (3.71–3.93)	3.44 (3.34–3.54)	10,167 (23.02%)	8.52 (8.33–8.72)	7.61 (7.44–7.79)	1,261,166,722
South	7,316 (37.09%)	3.14 (3.07–3.22)	2.95 (2.88–3.02)	14,989 (33.93%)	7.38 (7.24–7.52)	7.09 (6.96–7.22)	2,120,820,931
West	4,866 (24.67%)	3.34 (3.24–3.44)	3.37 (3.27–3.47)	7,963 (18.03%)	6.27 (6.11–6.44)	6.39 (6.22–6.56)	1,334,426,743

## Data Availability

The data used to support the findings of this are available from the corresponding author upon request.

## Abbreviation

RA: Rheumatoid arthritis
NVSS: National Vital Statistics System
WHO: The World Health Organization
CMR: Crude mortality rates
AAMR: Age-adjusted mortality rates
T1D: Type 1 diabetes
T2D: Type 2 diabetes.

## Supplementary Materials

Supplementary Materials The specific calculation method of US Standard population. Table S1: mortality from osteoporosis with or without diabetes according to year. Table S2: mortality from rheumatoid arthritis with or without diabetes according to year. Table S3: mortality from osteomyelitis with or without diabetes according to year.
