# Supplementary material for: The Characteristics and Mortality of Osteoporosis, Osteomyelitis, or Rheumatoid Arthritis in the Diabetes Population: A Retrospective Study
Source: Int J Endocrinol. 2020 Nov 7;2020:8821978. doi: 10.1155/2020/8821978 (PMC7669351; doi:10.1155/2020/8821978)
Supplement: Supplementary Materials — The specific calculation method of US Standard population. Table S1: mortality from osteoporosis with or without diabetes according to year. Table S2: mortality from rheumatoid arthritis with or without diabetes according to year. Table S3: mortality from osteomyelitis with or without diabetes according to year. [file 8821978.f1.zip › 8821978.f1/Supplement (1).docx]

Crude and age adjusted mortality incidence rates were calculated as deaths per 1,000,000 person-years. Each calculated age-specific death rate was multiplied by the standard population of that age interval, and this product was divided by the total standard population. Age-adjusted mortality rates and 95% confidence intervals were based on the following formula by the CDC WONDER system.

*R' =* $\int$*i (Psi / Ps) R i*

Where *i* is the age group, *R i* is the age-specific death rate, *Psi* is the standard population for age group *I*, and *Ps* is the total standard population. Risk ratios were calculated to compare mortality rates between subpopulations.

Standard error was calculated as follows:

S(R) = R *$\sqrt{1/D}$

Where R = death rate per 100,000, D = total number of deaths upon which the rate was based.

The 95% confidence intervals were calculated as follows:

- For ≥100 deaths: 95% CI: $R\pm1.96S(R)$;
- For <99 deaths: The upper/lower 95% confidence interval is the crude death multiplied by the upper/lower 95% confidence limit factor for a death rate based on a Poisson variable of the number of deaths.
