# Supplementary material for: The Characteristics and Mortality of Osteoporosis, Osteomyelitis, or Rheumatoid Arthritis in the Diabetes Population: A Retrospective Study
Source: Int J Endocrinol. 2020 Nov 7;2020:8821978. doi: 10.1155/2020/8821978 (PMC7669351; doi:10.1155/2020/8821978)
Supplement: Supplementary Materials — The specific calculation method of US Standard population. Table S1: mortality from osteoporosis with or without diabetes according to year. Table S2: mortality from rheumatoid arthritis with or without diabetes according to year. Table S3: mortality from osteomyelitis with or without diabetes according to year. [file 8821978.f1.zip › 8821978.f1/Table S1 (1).docx]

| Table S1. Mortality from osteoporosis with or without diabetes according to year | | | | | | | |
| --- | --- | --- | --- | --- | --- | --- | --- |
|  | **Both diabetes, osteoporosis, N (%)** | **Crude Rate Per 1,000,000** | **Age Adjusted Rate Per 1,000,000** | **Osteoporosis without diabetes, N (%)** | **Crude Rate Per 1,000,000** | **Age Adjusted Rate Per 1,000,000** | **Standard US Population in 2000** |
| Year |  |  |  |  |  |  |  |
| 1999 | 811 (4.40%) | 2.91  (2.71 - 3.11) | 2.98  (2.78 - 3.19) | 12,556(5.71%) | 45.00 (44.21 - 45.78) | 46.42 (45.61 - 47.23) | 279,040,168 |
| 2000 | 875 (4.75%) | 3.11  (2.90 - 3.32) | 3.18  (2.97 - 3.39) | 13,339(6.07%) | 47.40 (46.59 - 48.20) | 48.57 (47.74 - 49.39) | 281,421,906 |
| 2001 | 920 (4.99%) | 3.23  (3.02 - 3.44) | 3.29  (3.07 - 3.50) | 13,632(6.20%) | 47.84 (47.03 - 48.64) | 48.83 (48.01 - 49.65) | 284,968,955 |
| 2002 | 1,033 (5.61%) | 3.59  (3.37 - 3.81) | 3.63  (3.41 - 3.85) | 14,222(6.47%) | 49.45 (48.63 - 50.26) | 50.26 (49.44 - 51.09) | 287,625,193 |
| 2003 | 1,096 (5.95%) | 3.78  (3.55 - 4.00) | 3.79  (3.57 - 4.02) | 14,207(6.47%) | 48.97 (48.17 - 49.78) | 49.35 (48.54 - 50.16) | 290,107,933 |
| 2004 | 1,037 (5.63%) | 3.54  (3.33 - 3.76) | 3.56  (3.34 - 3.77) | 13,656(6.21%) | 46.64 (45.86 - 47.42) | 46.77 (45.98 - 47.55) | 292,805,298 |
| 2005 | 1,098 (5.96%) | 3.72  (3.50 - 3.94) | 3.69  (3.47 - 3.91) | 13,843(6.30%) | 46.84 (46.06 - 47.62) | 46.30 (45.52 - 47.07) | 295,516,599 |
| 2006 | 1,105 (6.00%) | 3.70  (3.48 - 3.92) | 3.63  (3.41 - 3.84) | 13,261(6.03%) | 44.44 (43.69 - 45.20) | 43.27 (42.53 - 44.01) | 298,379,912 |
| 2007 | 1,126 (6.11%) | 3.74  (3.52 - 3.96) | 3.64  (3.42 - 3.85) | 12,970(5.90%) | 43.06 (42.32 - 43.80) | 41.28 (40.57 - 41.99) | 301,231,207 |
| 2008 | 1,147 (6.22%) | 3.77  (3.55 - 3.99) | 3.61  (3.40 - 3.82) | 12,632(5.75%) | 41.54 (40.82 - 42.26) | 39.31 (38.62 - 40.00) | 304,093,966 |
| 2009 | 1,049 (5.69%) | 3.42  (3.21 - 3.63) | 3.23  (3.03 - 3.43) | 11,600(5.28%) | 37.81 (37.13 - 38.50) | 35.26 (34.61 - 35.90) | 306,771,529 |
| 2010 | 1,050 (5.70%) | 3.40  (3.20 - 3.61) | 3.21  (3.01 - 3.40) | 11,287(5.14%) | 36.56 (35.88 - 37.23) | 33.71 (33.09 - 34.34) | 308,745,538 |
| 2011 | 1,015 (5.51%) | 3.26  (3.06 - 3.46) | 2.98  (2.80 - 3.17) | 10,848(4.94%) | 34.81 (34.16 - 35.47) | 31.27 (30.68 - 31.86) | 311,591,917 |
| 2012 | 960 (5.21%) | 3.06  (2.86 - 3.25) | 2.76  (2.58 - 2.93) | 10,228(4.65%) | 32.58 (31.95 - 33.21) | 28.75 (28.19 - 29.31) | 313,914,040 |
| 2013 | 943 (5.12%) | 2.98  (2.79 - 3.17) | 2.60  (2.44 - 2.77) | 9,453(4.30%) | 29.90 (29.30 - 30.51) | 25.96 (25.43 - 26.49) | 316,128,839 |
| 2014 | 856 (4.56%) | 2.68  (2.50 - 2.86) | 2.32  (2.16 - 2.47) | 8,560(3.90%) | 26.85 (26.28 - 27.41) | 22.94 (22.45 - 23.43) | 318,857,056 |
| 2015 | 798 (4.33%) | 2.48  (2.31 - 2.66) | 2.12  (1.97 - 2.27) | 8,298(3.78%) | 25.82 (25.26 - 26.37) | 21.80 (21.33 - 22.28) | 321,418,820 |
| 2016 | 749 (4.06%) | 2.32  (2.15 - 2.48) | 1.95  (1.81 - 2.09) | 7,653(3.48%) | 23.68 (23.15 - 24.21) | 19.67 (19.23 - 20.11) | 323,127,513 |
| 2017 | 760 (4.12%) | 2.33  (2.17 - 2.50) | 1.92  (1.79 - 2.06) | 7,499(3.41%) | 23.02 (22.50 - 23.54) | 18.88 (18.45 - 19.31) | 325,719,178 |
