# Supplementary material for: The Characteristics and Mortality of Osteoporosis, Osteomyelitis, or Rheumatoid Arthritis in the Diabetes Population: A Retrospective Study
Source: Int J Endocrinol. 2020 Nov 7;2020:8821978. doi: 10.1155/2020/8821978 (PMC7669351; doi:10.1155/2020/8821978)
Supplement: Supplementary Materials — The specific calculation method of US Standard population. Table S1: mortality from osteoporosis with or without diabetes according to year. Table S2: mortality from rheumatoid arthritis with or without diabetes according to year. Table S3: mortality from osteomyelitis with or without diabetes according to year. [file 8821978.f1.zip › 8821978.f1/Table S2 (1).docx]

| Table S2. Mortality from rheumatoid arthritis with or without diabetes according to year | | | | | | | |
| --- | --- | --- | --- | --- | --- | --- | --- |
|  | **Both diabetes and rheumatoid arthritis, N (%)** | **Crude Rate Per 1,000,000** | **Age Adjusted Rate Per 1,000,000** | **Rheumatoid arthritis without diabetes, N (%)** | **Crude Rate Per 1,000,000** | **Age Adjusted Rate Per 1,000,000** | **Standard US Population in 2000** |
| Year |  |  |  |  |  |  |  |
| 1999 | 869 (4.22%) | 3.11 (2.91 - 3.32) | 3.18 (2.97 - 3.39) | 9,898(5.81%) | 35.47 (34.77 - 36.17) | 36.25 (35.53 - 36.96) | 279,040,168 |
| 2000 | 999 (4.85%) | 3.55 (3.33 - 3.77) | 3.61 (3.39 - 3.84) | 9,858(5.79%) | 35.03 (34.34 - 35.72) | 35.68 (34.98 - 36.39) | 281,421,906 |
| 2001 | 1,002 (4.87%) | 3.52 (3.30 - 3.73) | 3.56 (3.34 - 3.78) | 9,498(5.58%) | 33.33 (32.66 - 34.00) | 33.85 (33.17 - 34.53) | 284,968,955 |
| 2002 | 1,082 (5.26%) | 3.76 (3.54 - 3.99) | 3.80 (3.57 - 4.02) | 9,613(5.65%) | 33.42 (32.75 - 34.09) | 33.76 (33.09 - 34.44) | 287,625,193 |
| 2003 | 1,013 (4.92%) | 3.49 (3.28 - 3.71) | 3.49 (3.28 - 3.71) | 9,277(5.45%) | 31.98 (31.33 - 32.63) | 32.09 (31.44 - 32.75) | 290,107,933 |
| 2004 | 996 (4.84%) | 3.40 (3.19 - 3.61) | 3.40 (3.18 - 3.61) | 8,975(5.27%) | 30.65 (30.02 - 31.29) | 30.62 (29.98 - 31.25) | 292,805,298 |
| 2005 | 1,117 (5.43%) | 3.78 (3.56 - 4.00) | 3.75 (3.53 - 3.97) | 9,021(5.30%) | 30.53 (29.90 - 31.16) | 30.26 (29.63 - 30.88) | 295,516,599 |
| 2006 | 1,076 (5.23%) | 3.61 (3.39 - 3.82) | 3.53 (3.32 - 3.74) | 8,758(5.14%) | 29.35 (28.74 - 29.97) | 28.81 (28.21 - 29.42) | 298,379,912 |
| 2007 | 1,048 (5.09%) | 3.48 (3.27 - 3.69) | 3.37 (3.16 - 3.57) | 8,699(5.11%) | 28.88 (28.27 - 29.49) | 28.11 (27.51 - 28.70) | 301,231,207 |
| 2008 | 1,118 (5.43%) | 3.68 (3.46 - 3.89) | 3.53 (3.32 - 3.74) | 8,868(5.21%) | 29.16 (28.56 - 29.77) | 28.07 (27.48 - 28.66) | 304,093,966 |
| 2009 | 1,072 (5.21%) | 3.49 (3.29 - 3.70) | 3.33 (3.13 - 3.53) | 8,201(4.82%) | 26.73 (26.15 - 27.31) | 25.55 (24.99 - 26.10) | 306,771,529 |
| 2010 | 1,057 (5.14%) | 3.42 (3.22 - 3.63) | 3.22 (3.02 - 3.41) | 8,481 (4.98%) | 27.47 (26.88 - 28.05) | 25.91 (25.36 - 26.47) | 308,745,538 |
| 2011 | 1,179 (5.73%) | 3.78 (3.57 - 4.00) | 3.50 (3.30 - 3.70) | 8,839(5.19%) | 28.37 (27.78 - 28.96) | 26.30 (25.75 - 26.86) | 311,591,917 |
| 2012 | 1,155 (5.61%) | 3.68 (3.47 - 3.89) | 3.36 (3.16 - 3.55) | 8,721(5.12%) | 27.78 (27.20 - 28.36) | 25.37 (24.83 - 25.90) | 313,914,040 |
| 2013 | 1,140 (5.54%) | 3.61 (3.40 - 3.82) | 3.22 (3.03 - 3.41) | 8,729(5.13%) | 27.61 (27.03 - 28.19) | 24.77 (24.25 - 25.30) | 316,128,839 |
| 2014 | 1,102 (5.35%) | 3.46 (3.25 - 3.66) | 3.01 (2.83 - 3.19) | 8,579(5.04%) | 26.91 (26.34 - 27.47) | 23.70 (23.19 - 24.20) | 318,857,056 |
| 2015 | 1,153 (5.60%) | 3.59 (3.38 - 3.79) | 3.09 (2.91 - 3.27) | 8,575(5.04%) | 26.68 (26.11 - 27.24) | 23.17 (22.67 - 23.66) | 321,418,820 |
| 2016 | 1,151 (5.59%) | 3.56 (3.36 - 3.77) | 3.02 (2.85 - 3.20) | 8,759(5.14%) | 27.11 (26.54 - 27.67) | 23.09 (22.60 - 23.58) | 323,127,513 |
| 2017 | 1,255 (6.10%) | 3.85 (3.64 - 4.07) | 3.20 (3.02 - 3.38) | 8,942(5.25%) | 27.45 (26.88 - 28.02) | 23.00 (22.52 - 23.49) | 325,719,178 |
