# Supplementary material for: The Characteristics and Mortality of Osteoporosis, Osteomyelitis, or Rheumatoid Arthritis in the Diabetes Population: A Retrospective Study
Source: Int J Endocrinol. 2020 Nov 7;2020:8821978. doi: 10.1155/2020/8821978 (PMC7669351; doi:10.1155/2020/8821978)
Supplement: Supplementary Materials — The specific calculation method of US Standard population. Table S1: mortality from osteoporosis with or without diabetes according to year. Table S2: mortality from rheumatoid arthritis with or without diabetes according to year. Table S3: mortality from osteomyelitis with or without diabetes according to year. [file 8821978.f1.zip › 8821978.f1/Table S3 (1).docx]

| **Table S3. Mortality from osteomyelitis with or without diabetes according to year** | | | | | | | |
| --- | --- | --- | --- | --- | --- | --- | --- |
|  | **Both diabetes and osteomyelitis, N (%)** | **Crude Rate Per 1,000,000** | **Age Adjusted Rate Per 1,000,000** | **Osteomyelitis without diabetes, N (%)** | **Crude Rate Per 1,000,000** | **Age Adjusted Rate Per 1,000,000** | **Standard US**  **Population in 2000** |
| **Year** |  |  |  |  |  |  |  |
| 1999 | 722 (3.66%) | 2.59  (2.40 - 2.78) | 2.63  (2.44 - 2.82) | 1641 (3.72%) | 5.89  (5.54-6.23) | 6.07  (5.72-6.42) | 279,040,168 |
| 2000 | 731 (3.71%) | 2.60  (2.41 - 2.79) | 2.64  (2.45 - 2.84) | 1760 (3.98%) | 6.26  (6.07-6.78) | 6.39  (6.03-6.74) | 281,421,906 |
| 2001 | 803 (4.07%) | 2.82  (2.62 - 3.01) | 2.87  (2.67 - 3.07) | 1941 (4.39%) | 6.81  (6.45-7.17) | 6.91  (6.54-7.27) | 284,968,955 |
| 2002 | 857 (4.34%) | 2.98  (2.78 - 3.18) | 3.00  (2.79 - 3.20) | 1873 (4.24%) | 6.51  (6.16-6.87) | 6.57  (6.21-6.93) | 287,625,193 |
| 2003 | 906 (4.59%) | 3.12  (2.92 - 3.33) | 3.11  (2.91 - 3.31) | 1992 (4.51%) | 6.87  (6.51-7.24) | 6.88  (6.52-7.25) | 290,107,933 |
| 2004 | 972 (4.93%) | 3.32  (3.11 - 3.53) | 3.28  (3.07 - 3.48) | 2099 (4.75%) | 7.17  (6.80-7.54) | 7.15  (6.78-7.52) | 292,805,298 |
| 2005 | 906 (4.59%) | 3.07  (2.87 - 3.27) | 3.00  (2.81 - 3.20) | 2055 (4.65%) | 6.95  (6.59-7.31) | 6.87  (6.51-7.22) | 295,516,599 |
| 2006 | 935 (4.74%) | 3.13  (2.93 - 3.33) | 3.05  (2.85 - 3.24) | 2080 (4.71%) | 6.89  (6.62-7.34) | 6.76  (6.41-7.12) | 298,379,912 |
| 2007 | 946 (4.80%) | 3.14  (2.94 - 3.34) | 3.01  (2.82 - 3.20) | 2087 (4.72%) | 6.93  (6.57-7.29) | 6.66  (6.31-7.01) | 301,231,207 |
| 2008 | 895 (4.54%) | 2.94  (2.75 - 3.14) | 2.77  (2.59 - 2.96) | 2027 (4.59%) | 6.67  (6.32-7.02) | 6.36  (6.02-6.69) | 304,093,966 |
| 2009 | 912 (4.62%) | 2.97  (2.78 - 3.17) | 2.78  (2.60 - 2.97) | 2134 (4.83%) | 6.96  (6.61-7.32) | 6.53  (6.19-6.86) | 306,771,529 |
| 2010 | 961 (4.87%) | 3.11  (2.92 - 3.31) | 2.90  (2.71 - 3.08) | 2224 (5.04%) | 7.21  (6.85-7.56) | 6.68  (6.35-7.02) | 308,745,538 |
| 2011 | 1030 (5.22%) | 3.31  (3.10 - 3.51) | 3.01  (2.82 - 3.19) | 2332 (5.28%) | 7.48  (7.12-7.84) | 6.84  (6.51-7.18) | 311,591,917 |
| 2012 | 1059 (5.37%) | 3.37  (3.17 - 3.58) | 3.01  (2.83 - 3.19) | 2479 (5.61%) | 7.90  (7.53-8.27) | 7.14  (6.9-7.48) | 313,914,040 |
| 2013 | 1177 (5.97%) | 3.72  (3.51 - 3.94) | 3.25  (3.06 - 3.44) | 2620 (5.93%) | 8.29  (7.91-8.67) | 7.3  (6.96-7.64) | 316,128,839 |
| 2014 | 1242 (6.30%) | 3.90  (3.68 - 4.11) | 3.37  (3.18 - 3.56) | 2796 (6.33%) | 8.76  (8.37-9.15) | 7.62  (7.28-7.97) | 318,857,056 |
| 2015 | 1474 (7.47%) | 4.59  (4.35 - 4.82) | 3.93  (3.72 - 4.13) | 3099 (7.02%) | 9.64  (9.23-10.05) | 8.27  (7.91-8.63) | 321,418,820 |
| 2016 | 1518 (7.70%) | 4.70  (4.46 - 4.93) | 3.98  (3.78 - 4.18) | 3302 (7.48%) | 10.22  (9.80-10.64) | 8.70  (8.34-9.07) | 323,127,513 |
| 2017 | 1680 (8.52%) | 5.16  (4.91 - 5.40) | 4.25  (4.05 - 4.46) | 3630 (8.22%) | 11.14  (10.7-11.58) | 9.29  (8.92-9.66) | 325,719,178 |
